# The Association between Dietary Inflammatory Patterns and the Incidence of Frailty and Its Reversal in Older Adults: A Community-Based Longitudinal Follow-Up Study in Taiwan

**DOI:** 10.3390/nu16172862

**Published:** 2024-08-27

**Authors:** Shu-Chun Chuang, Chao A. Hsiung, Meng-Hua Tao, I-Chien Wu, Chiu-Wen Cheng, Wei-Ting Tseng, Marion M. Lee, Hsing-Yi Chang, Chih-Cheng Hsu

**Affiliations:** 1 Institute of Population Health Sciences, National Health Research Institutes, Zhunan, Miaoli 350401, Taiwanicwu@nhri.edu.tw (I.-C.W.); chiuwen@nhri.edu.tw (C.-W.C.); weiting@nhri.edu.tw (W.-T.T.); hsingyi@nhri.edu.tw (H.-Y.C.); cch@nhri.edu.tw (C.-C.H.); 2 Department of Public Health Sciences, Henry Ford Health, Detroit, MI 48202, USA; mtao1@hfhs.org; 3 Department of Epidemiology and Biostatistics, University of California San Francisco, San Francisco, CA 94143, USA; marion.lee@ucsf.edu; 4 National Center for Geriatrics and Welfare Research, National Health Research Institutes, Yunlin 632007, Taiwan

**Keywords:** dietary inflammatory pattern, frailty, aging

## Abstract

Dietary patterns related to inflammation have garnered great interest in disease prevention. The aims of this study were to evaluate whether a proinflammatory diet affects the incidence of frailty and its reversal in a prospective follow-up study. Data were taken from 5663 community-dwelling individuals ≥ 55 years old in Taiwan. The energy-adjusted dietary inflammatory index (DII) and the Empirical Dietary Inflammatory Patterns-Healthy Aging Longitudinal Study in Taiwan (EDIP-HALT) at baseline were calculated using a food frequency questionnaire. Frailty was assessed with Fried’s criteria in 2008–2013 and 2013–2020. Associations with changes in frailty status were assessed with multinominal logistic regressions and adjusted for major confounders. Higher EDIP-HALST scores (proinflammatory) were associated with higher odds of frailty among baseline robust participants in men (OR = 2.44, 95% CI = 1.42–4.21, *p-*_trend_ < 0.01) and broadline associated in women (OR = 1.96, 95% CI = 0.96–3.98, *p-*_trend_ = 0.05), but associated with lower odds of reversing back to robust among baseline prefrail participants. However, the later association was only observed in women, and the relationships were stronger in the middle tertile (second vs. first tertile, OR = 0.40, 95% CI = 0.25–0.65). A pro-inflammatory diet pattern was associated with higher odds of frailty onset in baseline robust participants and lower odds of reversal in baseline prefrail female participants.

## 1. Introduction

Frailty is a syndrome or condition that reflects an individual’s vulnerability to stressors that increase the risks of adverse health outcomes, such as dementia, disability, hospitalization, and death [1,2]. Fortunately, frailty is reversible. Identifying individuals at high risk for adverse health outcomes, such as frailty, and developing upstream interventions might prevent disease progression and promote a healthy lifespan. Dietary habits are modifiable and related to several age-related chronic diseases, making diet an applicable strategy for disease prevention interventions.

Several pieces of research have been conducted on known dietary patterns and frailty risk, including the Mediterranean diet [3], Nordic diet [4], and plant-based diets [5]. Recently, innovative methodological approaches were also used to explore the empirical dietary patterns and frailty risks, such as factor analysis [6], reduced rank regression (RRR) [7], and machine learning [8]. However, these previous studies were based on known healthy diets or purely data-driven. Nevertheless, a dietary pattern that targets disease etiology might also benefit the development of a disease prevention strategy.

Inflammation is a common phenomenon that is involved in almost all chronic conditions, including musculoskeletal health and frailty [9,10,11]. Inflammation accelerates the aging process and initiates age-related diseases [12,13]. A dietary pattern with inflammatory properties might be associated with inflammatory-related health conditions, for example, frailty. Several dietary indices have been created to evaluate the inflammatory potential of diets [14], such as the Dietary Inflammatory Index (DII) developed by Shivappa et al. [15] and the Empirical Dietary Inflammatory Pattern (EDIP) by Tabung et al. [16]. Previous studies have shown that high DII scores are associated with musculoskeletal health [17], sarcopenia [18,19], and frailty [20,21,22,23,24]. However, the results for EDIP were less consistent [20,25]. Because of the reproducibility issue of EDIP in different populations with different cultures [25,26,27], we followed the methods described in Tabung et al. [16] and created the EDIP-Healthy Longitudinal Study in Taiwan (HALST) in our previous work [28].

The aim of this study was to evaluate the association between a dietary inflammatory pattern, as assessed by EDIP-HALST, and changes in physical performance and frailty status in a community-based longitudinal follow-up study in Taiwan.

## 2. Material and Methods

### 2.1. The Healthy Aging Longitudinal Study in Taiwan (HALST)

The HALST study is prospective research of community-dwelling older adults that recruited 5663 volunteers across Taiwan between 2008 and 2013. The cohort has been previously described [29]. In brief, all eligible residents (≥55 years old) living within the catchment area of seven collaborative hospitals were selected for the study. Participants with any of the following conditions were excluded: highly contagious infectious diseases, diagnosed dementia, severe illness, being bedridden, severe mental disorder, mutism, hearing impairment, blindness, living in a long-term care facility, or being hospitalized. The original response rate was 31%. Interviewers were trained to conduct face-to-face interviews using the participant’s native language, including Taiwanese and Hakka. Among the 5663 participants, 79 were excluded due to unreliable energy intake.

The second wave of data collection was initiated in 2013 and completed in 2020. Between the two assessments, 606 participants died, 137 were too ill to participate, 515 refused to participate in the second wave of data collection, 213 were not able to be re-contacted, and 4113 participants (73.7%) completed the follow-up assessment (Supplementary Figure S1). The median time between baseline and the second wave of data collection was 6.4 years (range 4.4–11.4 years). The participants who stayed in the study tended to be younger and had a higher education level than those who dropped out (Supplementary Table S1).

Written informed consent was provided by each participant at baseline and follow-up. The study was approved by the institutional review board of the National Health Research Institutes and the collaborative hospitals (EC0970608, EC1020805, EC1081102). All methods were performed in accordance with the relevant guidelines and regulations.

### 2.2. Description of the Dietary Assessment in the HALST

All participants were asked to complete a 72-item food frequency questionnaire (FFQ) to estimate the average daily dietary intake over the past year. The original FFQ was developed for Chinese Americans and subsequently adapted for a validation study in the Taiwanese population [30,31], which suggests that this FFQ should provide reasonable estimates of typical intake in epidemiologic studies in Taiwan [30,31]. Average consumptions (g/day) were calculated from the frequency of eating and portion size. Energy and nutrient intakes were calculated using the Food Composition Tables in Taiwan (released in March 2017, the earliest version that was publically available when the dietary data were processed). The energy and nutrient intakes were calculated as the sum of the product of the frequency of eating, portion size, and energy and nutrient content in each food referenced from the Food Composition Tables. Energy intake >5000 kcal for men, >4500 kcal for women, or <500 kcal for both men and women were deleted from the data (*n* = 79).

#### 2.2.1. Description of the EDIP-HALST Score

The development and validation of the EDIP-HALST had been previously described [28]. Briefly, the HALST study participants were divided into exploratory and confirmatory samples. Using data from the exploratory sample, intakes of the 27 previously defined food groups were calculated and applied to the reduced rank regression (RRR) models to derive a dietary pattern predictive of three inflammatory biomarkers: high-sensitivity C-reactive protein (hs-CRP), tumor necrosis factor receptor 1 (TNFR1), and interleukin-6 (IL-6). Then, the first factor obtained via the RRR was used for further data reduction in stepwise linear regression analyses to identify the most important food groups that contributed to the dietary pattern. The factor obtained from the RRR was treated as the dependent variable, and the 27 food groups were the independent variables. The significance level for entering and staying in the model was set to *p* = 0.1. The regression coefficients from the last step of the stepwise linear regression model were used as the component weights. The validity of the EDIP-HALST score was evaluated in the confirmatory sample.

#### 2.2.2. Description of Energy-Adjusted DII

As a comparison, the energy-adjusted DII was calculated according to Shivappa et al. [15,32]. Briefly, 22 of the possible 45 items were standardized using the world averages and standard deviations (SDs) provided in the publication [15]. The 22 nutrients available in the current study were: alcohol, vitamin B_12_, vitamin B_6_, beta-carotene, carbohydrate, cholesterol, total fat, fiber, folate, iron, magnesium, monounsaturated fatty acid, niacin, protein, polyunsaturated fat, riboflavin, saturated fat, thiamin, vitamin A, vitamin C, vitamin E, and Zinc. The values were converted to centered proportion scores, which were then multiplied by their respective inflammatory effect score and added to obtain an overall DII for each individual. Because total energy intake influences overall nutrient intake, the DII was calculated using the residual method [32]. A higher DII score indicates a more pro-inflammatory dietary component.

### 2.3. Assessment of Physical Performance

The physical performance was measured for handgrip strength and Short Physical Performance Battery (SPPB) during the home visit.

The handgrip strength was measured with the North Coast Hand Dynamometer (North Coast Medical Inc., Gilroy, CA, USA). The analysis was based on the best performance (kg) of the three trials in the dominant hand.

The SPPB, which included gait speed, five-times chair stands, and standing balance, was scored by the following criteria [33]. For tests of gait speed, the faster of the two walks was used to define the scores: 0 if a participant could not perform the test, 1 if his/her speed was ≤0.43 m/s, 2 if 0.44–0.60 m/s, 3 if 0.61–0.77 m/s, and 4 if >0.77 m/s. For five-timed chair stands, the scores were defined as follows: 0 if a participant was unable to perform the test, 1 if he/she completed the test in >16.7, score 2 if 16.6–13.7, 3 if 13.6–11.2, and 4 if ≤11.1 s. For standing balance, we asked the participants to maintain balance in three different standing positions for at least 10 s. A participant was awarded 0 points for failing to perform the test, 1 point for standing side-by-side for 10 s but unable to hold a semi-tandem stance for 10 s, 2 points for standing semi-tandem for 10 s but unable to hold a full-tandem stance for more than 2 s, 3 points for standing full-tandem for 3–9 s, and 4 points for standing full-tandem for 10 s. A composite performance score was the sum of the scores calculated on the three tests, with 0 being the worst and 12 being the best.

Poor physical performances were defined as follows: handgrip strength < 28 kg for men and <17 kg for women (lower sex-specific 20% based on baseline measurements), gait speed ≤ 0.43 m/s, five-timed chair stands >13.6 s, failure to hold a full-tandem stance for 10 s, and SPPB < 10.

### 2.4. Definition of Physical Frailty

The definition of physical frailty was modified by Fried et al. [34]. Briefly, the following five phenotypes of physical conditions were defined from the questionnaire or the measured physical performance: “weight loss” as a self-reported involuntary loss of more than 4.5 kg body weight during the past year; “exhaustion” as a self-response of “a moderate amount of the time” or “most of the time” to either of the following statements: “I felt that everything I did was an effort” or “I could not get going”; “low physical activity” as the lower 20% sex-specific physical activity (kcal/week); “slow walking speed” as the lower 20% sex- and height-specific cutoffs of the walking speed in the 3- or 4 m walking test or unable to perform the test; and “weakness” as the lower 20% sex- and body-mass-index (BMI)-specific cutoffs in the handgrip strength test. Participants without any of these conditions were considered physically robust, pre-frail if they had one or two conditions, and frail if they had three or more conditions.

### 2.5. Ascertainment of Vital Status

The vital status, causes, and date of death of the participants were confirmed via data linkage with the Taiwan National Death Certificate database. To minimize the attrition due to death in the follow-up assessment, if a participant died before the last date of recruitment date of the follow-up, he/she was included in the frail group.

### 2.6. Measurement of Covariates

A number of covariates, including age, sex, education level (low literacy, primary school, or more than primary school), smoking, height, weight (for calculating BMI), waist circumference, personal history of medical conditions (including diabetes, heart disease, stroke, hyperlipidemia, asthma, chronic respiratory tract disease, cancer, gastric disease, liver and gallbladder disease, cataract, gout, anemia, kidney diseases, arthritis, spurs, osteoporosis, fracture, and mental illness), social networking score and Center for Epidemiologic Studies—Depression Scale (CES-D) scores, were selected as potential confounders from the literature [35,36]. The study center was included as a surrogate for urbanization. Most of the covariates were self-reported, with the exception of height and weight, which were measured during the physical examination by the trained interviewers.

### 2.7. Statistical Analysis

The participants’ characteristics were expressed as count (%) and compared using the chi-square test for categorical variables or described by means ± SDs and compared by the Kruskal–Wallis test for continuous variables. Data from the developing and validating samples were pooled for analysis of the association between dietary scores and physical performance and frailty. Tertiles of each dietary score were categorized according to the distribution by sex. The Poisson regression models with robust error variance [37] were used to estimate relative risks (RR) and 95% confidence intervals (CI) for poor physical performance, with the lowest tertile of the dietary scores serving as the reference category. To properly evaluate the associations with changes in physical performance from baseline to follow-up, participants with poor physical performance at baseline were excluded from the respective analysis. The multinominal logistic regression models were used to analyze the odds ratios (OR) and 95% CI for the association between dietary scores and frailty at follow-up. The analyses were separated by sex and adjusted for age at recruitment (continuous), center, education (illiterate, elementary school, middle school and above), smoking status (never, former, and current), physical activity at leisure time and at work (sex-specific tertiles), BMI (<18.5, 18.5–25, 25–30, and ≥30 kg/m^2^), and number of chronic diseases (0–2, 3–5, and ≥6), social networking score and CES-D scores (<16 or ≥16). For associations with physical frailty, physical activity, and CES-D were removed from adjustment because part of the two variables were used in frailty definition. Confounders were selected based on a literature review. The *p*-_trend_ was obtained by using the continuous dietary scores adjusted to the covariates.

All analyses were performed using SAS 9.4. Statistical significance was defined as α < 0.05.

## 3. Results

The characteristics of the participants by tertile of the EDIP-HALST scores are presented in Table 1. Overall, men and women with higher EDIP-HALST scores were associated with higher age, lower education, lower leisure-time physical activity, lower social network scores, and higher prevalence of depression. In women, higher EDIP-HALST scores were also associated with higher work-related physical activity.

Table 2 shows the association between baseline dietary inflammatory scores and poor physical performance at follow-up. Interestingly, EDIP-HALST scores were associated with higher risk of poor handgrip strength in women (third vs. first tertile, RR = 1.46, 95% CI = 1.10–1.96, *p-*_trend_ < 0.01 for EDIP-HALST) and poor lower limb strength (RR = 1.70, 95% CI = 1.03–2.80, *p-*_trend_ = 0.04 for poor five-timed chair stands and RR = 1.47, 95% CI = 1.03–2.10, *p-*_trend_ = 0.05 for poor SPPB) in men.

The association between these dietary scores and change in frailty status is shown in Table 3. Higher EDIP-HALST scores were associated with higher odds of frailty among baseline robust participants in men (OR = 2.44, 95% CI = 1.42–4.21, *p-*_trend_ < 0.01) and broadline associated in women (OR = 1.96, 95% CI = 0.96–3.98, *p-*_trend_ = 0.05), but associated with lower odds of reversing back to robust among baseline prefrail participants. However, the later association was only observed in women, and the relationships were stronger in the middle tertile (second vs. first tertile, OR = 0.40, 95% CI = 0.25–0.65).

## 4. Conclusions

In this study, we evaluated the association between a dietary score that may reflect the inflammatory potential of the Taiwanese diet and physical performance and frailty. The score was related to a higher risk of poor handgrip strength in women and five-timed chair stands in men, higher odds of prefrail and frail onset in baseline robust participants, and lower odds of reversing back to robust in baseline prefrail female participants.

Population-based studies have indicated that chronic systemic inflammation is directly involved in muscle catabolism [11], and increased levels of IL-6, TNF-α, and CRP are related to loss of muscle mass, reduced muscle strength, and poor overall muscle function [38,39,40]. Diet can modulate microflora populations in the intestinal lumen and hence may influence the immune response and disease risk [41,42,43]. More specifically, a United States microbiome study found that DII is associated with *Ruminococcus torques*, *Eubacterium nodatum*, and *Acidaminococcus intestini* [44], and a European study revealed that both DII and EDIP explain lipoperoxidation levels [45]. DII has been associated with several age-related phenotypes, including falls [46], musculoskeletal health [17,47,48], sarcopenia [18,19], and frailty [20,21,22,23,24,49]. These microbiota studies further suggest that diet can modulate the inflammatory environment by altering gut microbiota and metabolites, making dietary inflammatory scores a promising strategy for diet intervention. 

However, the influence of an inflammatory environment on muscle health seemed to be sex-specific, as we observed an association of a pro-inflammatory diet with poor handgrip strength in women and poor lower limb strength in men. Although EDIP-HALST was associated with the onset of frailty in both men and women, the inverse association with reversing prefrail status was only observed in women. Research from the English Longitudinal Study of Aging found that lower levels of CRP and fibrinogen were associated with higher handgrip strength in women but not men [50]. Previous studies have also shown that the inflammatory profile is different in men and women with frailty and sarcopenia [51,52]. For example, the association between TNF-α and chair stands is positive in men but negative in women, and the association between CRP and handgrip strength, gait speed, and SPPB is negative in men but positive in women [51]. Previous studies suggested that estrogen is important for muscle mass maintenance [53]. Women after menopause may be particularly more susceptible to inflammation-mediated muscle loss. The effect of reducing inflammatory stress via diet might also be sex-specific. Additional studies are needed to tailor dietary interventions for physical performance and frailty.

By contrast, there has been less research on associations between EDIP and frailty. A study in Spain suggested that the DII but not EDIP predicted frailty and disability in their population [20]. The authors speculated that the population-dependent EDIP biomarker association may further impede its application in assessing diet and disease associations in the Spanish population [20]. A Brazilian research group also showed that the EDIP for the Sao Paulo population, which included processed meats, fruits and vegetables, and rice and beans [25], outperformed the original version of EDIPs that was validated in US populations [16,54,55]. We also demonstrated that the EDIP-HALST outperformed the DII from Shivappa’s algorithm in our previous work [28]. Due to different cultures and eating habits, variations in inflammatory dietary patterns in different populations are expected. Taiwanese are a rice-eating population. Choosing whole-grain rice and more fruit and vegetables and avoiding refined-grain rice is associated with lower inflammation in both men and women and should be encouraged.

In addition to diet, other lifestyles may also contribute to inflammation and frailty, such as smoking. In our study, the prevalence of smoking was higher in men than in women (25% vs. 2%), which might be another explanation for why the associations are stronger and more consistent in women than in men. Additional studies are needed in men to confirm these findings.

In addition to inflammatory properties, other dietary properties, such as protein intake, may also play a role in frailty [56]. However, protein is considered pro-inflammatory, as measured by the DII [15]. Another Taiwanese study suggested that dietary patterns with more plant-based foods, tea, fish, shellfish, and milk, are related to a lower prevalence of frailty [7]. An Italian study identified dietary profiling of physical frailty using LASSO regression and found that higher intakes of legumes and lower consumption of wine and coffee were associated with a higher risk of frailty [8]. In contrast to these previous studies that directly investigated frailty-related dietary patterns, we studied the inflammatory potential of dietary patterns and evaluated whether these patterns are related to frailty. The results of our study provide additional evidence of the potential of preventing inflammatory-related diseases with healthy dietary patterns.

This study had some limitations. First, the FFQ is prone to response bias and measurement errors [57]. Nevertheless, the FFQ has been found to have good repeatability for most nutrients in older adults [58]. Furthermore, misclassification in dietary inflammatory patterns due to misreporting may be non-differential; thus, attenuated associations are expected. Second, only 22 of the 45 items for evaluating DII were available in our dietary data. The range of DII in the current study was only −4.68–4.37, which is narrower than its plausible range −8.87–7.98 [15]. However, previous studies have suggested that the range of DII scores relies on the amount of food intake rather than the number of DII components available [59], and the predictability of DII remains the same when the number of DII components is reduced to 28 [60]. Finally, the EDIP-HALST scores were only validated within our own cohort. These scores should be evaluated with a wider spectrum of inflammatory biomarkers and validated in other Taiwanese populations.

The major strength of this study is its prospective study design, which enabled us to observe physical changes from baseline. In addition, all participants in our cohort were followed by trained interviewers to ensure homogeneity and objectivity, thus reducing the information bias. Moreover, our cohort included a relatively large group of community-dwelling participants who were able to provide detailed data about other covariates, such as education, lifestyle, smoking behavior, comorbidities, medications, depression, and social network, thereby enabling us to explore associations independent of these covariates.

In summary, our results suggest that a proinflammatory dietary pattern, as measured by DII and EDIP-HALST scores, is associated with higher odds of developing frailty in baseline physically robust participants and lower odds of reversing back to robust in female prefrail participants. More studies are needed to evaluate the impact of dietary inflammatory patterns on preventing the onset of frailty and reversing the frailty status.

## Figures and Tables

**Table 1:** Participant characteristics at baseline by tertile of the EDIP-HALST scores.

	Men							Women						
	T1		T2		T3			T1		T2		T3		
	Mean	SD	Mean	SD	Mean	SD	*p*	Mean	SD	Mean	SD	Mean	SD	*p*
Age	67.65	7.66	70.51	8.50	71.79	8.76	<0.01	68.17	7.86	69.49	7.94	70.42	3.06	<0.01
Body mass index	24.64	3.16	24.52	3.27	24.33	3.48	0.07	24.27	3.57	24.76	3.69	24.70	3.72	<0.01
Abdominal circumference	87.12	9.09	87.29	8.94	87.30	9.83	0.92	84.49	10.93	86.22	11.04	86.16	11.01	<0.01
Energy intake	2435.66	788.94	2150.27	717.54	2427.47	870.59	<0.01	1892.46	665.70	1673.67	602.84	1979.11	640.39	<0.01
	N	%	N	%	N	%		N	%	N	%	N	%	
Education levels														
Illiteracy	15	1.7	32	3.7	41	4.7	<0.01	68	6.9	181	18.4	275	27.9	<0.01
Primary school	276	31.6	381	43.6	385	44.0		414	42.2	516	52.3	508	51.5	
More than primary school	582	66.7	461	52.7	449	51.3		501	50.9	289	29.3	203	20.6	
Missing	1		1		0			2		0		0		
Smoking														
Never	396	45.3	356	40.7	366	41.8	0.02	972	98.7	960	97.4	957	97.1	0.06
Former	246	28.1	309	35.3	300	34.3		4	0.4	11	1.1	7	0.7	
Current	232	26.5	210	24.0	209	23.9		9	0.9	15	1.5	22	2.2	
Physical activity in leisure time (sex-specific tertile)														
Low	233	26.7	307	35.1	330	38.0	<0.01	207	21.1	346	35.4	427	43.4	<0.01
Median	289	33.1	290	33.2	294	33.8		338	34.5	329	33.7	313	31.8	
High	350	40.1	277	31.7	245	28.2		435	44.4	302	30.9	243	24.7	
Missing	2		1		6			5		9		3		
Physical activity at work (sex-specific median)														
No	670	76.7	633	72.5	653	74.6	0.08	802	81.4	729	74.5	687	70.1	<0.01
Low	116	13.3	123	14.1	104	11.9		115	11.7	126	12.9	145	14.8	
High	87	10.0	117	13.4	118	13.5		68	6.9	124	12.7	148	15.1	
Missing	1		2		0			0		7		6		
Number of chronic diseases														
0–2	397	45.4	396	45.3	371	42.4	0.02	380	38.6	383	38.8	366	37.1	0.19
3–5	381	43.6	369	42.2	361	41.3		446	45.3	435	44.1	482	48.9	
≥6	96	11.0	110	12.6	143	16.3		159	16.1	168	17.0	138	14.0	
Social network														
≥8	435	49.8	443	50.6	383	43.8	0.04	504	51.2	520	52.7	421	42.7	<0.01
6–7	234	26.8	230	26.3	259	29.6		267	27.1	240	24.3	319	32.4	
0–5	205	23.5	202	23.1	233	26.6		214	21.7	226	22.9	246	24.9	
CESD														
<16	853	97.6	836	95.7	834	95.4	0.03	935	94.9	913	92.6	896	90.9	<0.01
≥16	21	2.4	38	4.3	40	4.6		50	5.1	73	7.4	90	9.1	
Missing	0		1		1			0		0		0		

CESD: Center for Epidemiologic Studies Depression Scale; EDIP-HALST: Empirical Dietary Inflammatory Pattern-Healthy Aging Longitudinal Study in Taiwan; SD: Standard deviation.

**Table 2:** Association between baseline dietary inflammatory scores and poor physical performance at follow-up ^1^.

	Men						Women					
	T1	T2		T3			T1	T2		T3		
	RR	RR^2^	95% CI	RR ^2^	95% CI	*p*_-trend_ ^3^	RR	RR ^2^	95% CI	RR ^2^	95% CI	*p*_-trend_ ^3^
Grip strength < 20% (M = 1645/F = 1796)												
DII	1.00	0.96	(0.74, 1.24)	0.95	(0.73, 1.23)	0.61	1.00	1.59	(1.19, 2.13)	1.57	(1.16, 2.13)	0.06
EDIP-HALST	1.00	1.04	(0.80, 1.35)	1.08	(0.83, 1.40)	0.48	1.00	1.58	(1.20, 2.08)	1.46	(1.10, 1.96)	<0.01
Succeed in full tandem stands (M = 1717/F = 1824)												
eDII	1.00	1.05	(0.82, 1.35)	1.07	(0.83, 1.39)	0.66	1.00	1.12	(0.92, 1.37)	1.11	(0.91, 1.36)	0.64
EDIP-HALST	1.00	1.05	(0.82, 1.36)	1.03	(0.80, 1.34)	1.00	1.00	1.03	(0.85, 1.26)	1.01	(0.82, 1.23)	0.75
Gait speed ≤ 0.6 m/s (M = 1723/F = 1824)												
DII	1.00	1.13	(0.74, 1.71)	1.36	(0.92, 2.01)	0.13	1.00	0.82	(0.57, 1.18)	0.99	(0.71, 1.39)	0.84
EDIP-HALST	1.00	0.94	(0.62, 1.45)	1.45	(0.98, 2.15)	0.07	1.00	1.06	(0.75, 1.49)	0.85	(0.60, 1.19)	0.86
5-timed chair stands >13.6 s (M = 1674/F = 1721)												
DII	1.00	1.22	(0.73, 2.06)	1.31	(0.77, 2.21)	0.51	1.00	1.24	(0.84, 1.84)	1.31	(0.90. 1.92)	0.11
EDIP-HALST	1.00	0.84	(0.48, 1.45)	1.70	(1.03, 2.80)	0.04	1.00	1.32	(0.90, 1.93)	1.23	(0.83, 1.83)	0.47
SPPB < 10 (M = 1615/F = 1626)												
DII	1.00	1.21	(0.83, 1.75)	1.21	(0.84, 1.75)	0.48	1.00	1.01	(0.73, 1.39)	1.24	(0.94, 1.66)	0.16
EDIP-HALST	1.00	1.08	(0.75, 1.56)	1.47	(1.03, 2.10)	0.05	1.00	1.19	(0.88, 1.59)	1.03	(0.76, 1.39)	0.54

^1^. Poor physical performance at baseline was excluded. ^2^. Poisson regression adjusted for age at recruitment (continuous), center, education (illiteracy, primary school, middle school and above), smoking status (never, former, and current), physical activity at leisure time and at work (sex-specific tertile), body mass index (<18.5, 18.5–25, 25–30, and ≥30 kg/m^2^), number of chronic diseases (0–2, 3–5, and ≥6), social network score, and CES-D scores (<16 or ≥16). ^3^. The *p*-value for linear trend was the *p*-value of the dietary score as a continuous variable adjusted for all covariates listed in footnote ^2^. CI: Confidence interval; DII: Dietary Inflammatory Index; EDIP-HALST: Empirical Dietary Inflammatory Pattern-Healthy Aging Longitudinal Study in Taiwan; RR: Relative risk; SPPB: Short Physical Performance Battery.

**Table 3:** Association between baseline dietary inflammatory scores and change in frailty status.

Frailty at Baseline	Robust				Pre-Frail			
Frailty at Follow-Up	Prefrail vs. Robust		Frail/Death vs. Robust		Robust vs. Prefrail		Frail/Death vs. Prefrail	
Men	OR	95% CI	OR	95% CI	OR	95% CI	OR	95% CI
DII								
T1	1.00		1.00		1.00		1.00	
T2	1.30	(0.93, 1.82)	2.21	(1.33, 3.69)	0.95	(0.56, 1.62)	1.50	(0.95, 2.38)
T3	1.14	(0.79, 1.63)	1.35	(0.77, 2.34)	0.73	(0.42, 1.25)	1.23	(0.78, 1.95)
*p*_-trend_	0.28		0.59		0.14		0.75	
EDIP-HALST								
T1	1.00		1.00		1.00		1.00	
T2	0.94	(0.67, 1.31)	2.00	(1.18, 3.37)	0.68	(0.40, 1.15)	0.79	(0.50, 1.23)
T3	1.33	(0.94, 1.88)	2.44	(1.42, 4.21)	1.19	(0.72, 1.99)	1.10	(0.70, 1.72)
*p*_-trend_	0.04		<0.01		0.29		0.47	
Women								
DII								
T1	1.00		1.00		1.00		1.00	
T2	1.46	(1.03, 2.05)	2.03	(0.97, 4.26)	0.39	(0.24, 0.64)	0.82	(0.47, 1.42)
T3	1.79	(1.25, 2.56)	3.46	(1.69, 7.09)	0.51	(0.31, 0.84)	0.96	(0.55, 1.68)
*p*_-trend_	0.01		<0.01		0.07		0.67	
EDIP-HALST								
T1	1.00		1.00		1.00		1.00	
T2	1.14	(0.81, 1.62)	1.64	(0.81, 3.31)	0.40	(0.25, 0.65)	0.77	(0.45, 1.33)
T3	1.23	(0.85, 1.77)	1.96	(0.96, 3.98)	0.66	(0.40, 1.07)	1.12	(0.65, 1.96)
*p*_-trend_	0.24		0.05		0.01		0.57	

The ORs were adjusted for age at recruitment (continuous), center, education (illiteracy, primary school, middle school, and above), smoking status (never, former, and current), body mass index (BMI, <18.5, 18.5–25, 25–30, and ≥30 kg/m^2^), number of chronic diseases (0–2, 3–5, and ≥6), and social network score. CI: Confidence interval; DII: Dietary Inflammatory Index; EDIP-HALST: Empirical Dietary Inflammatory Pattern-Healthy Aging Longitudinal Study in Taiwan; OR: Odds ratio.

## Data Availability

The dataset is available upon reasonable request to the corresponding author. The data are not publicly available due to privacy.

## Supplementary Materials

The following supporting information can be downloaded at: https://www.mdpi.com/article/10.3390/nu16172862/s1, Figure S1: Baseline characteristics for participants included and not included in the follow-up; Table S1: Flow chart of data for analysis.

## Abbreviations

BMI: Body mass index; CI: Confidence interval; DII: Dietary Inflammatory Index; EDIP: Empirical Dietary Inflammatory Pattern; FFQ: Food frequency questionnaire; HALST: Healthy Aging Longitudinal Study in Taiwan; hs-CRP: High-sensitivity C-reactive protein; IL-6: Interleukin-6; OR: Odds ratio; RR: Relative risk RRR: Reduced rank regression; SD: Standard division; SPPB: Short Physical Performance Battery; TNFR1: Tumor necrosis factor receptor 1.
