# Supplementary material for: The Association between Dietary Inflammatory Patterns and the Incidence of Frailty and Its Reversal in Older Adults: A Community-Based Longitudinal Follow-Up Study in Taiwan
Source: Nutrients. 2024 Aug 27;16(17):2862. doi: 10.3390/nu16172862 (PMC11397639; doi:10.3390/nu16172862)

Supplementary Table S1. Baseline characteristics for participants included and not included in the follow-up

	Complete		Death or too ill to participate		Refuse or withdraw		
	N	%	N	%	N	%	p
**Age**							
55-65	1399	34.0	61	8.2	191	26.2	<0.01
65-75	1898	46.2	242	32.6	326	44.8	
≥75	816	19.8	440	59.2	211	29.0	
**Sex**							
Men	1913	46.5	443	59.6	271	37.2	<0.01
Women	2200	53.5	300	40.4	457	62.8	
**Education**							
Low literacy	353	8.6	145	19.6	114	15.7	<0.01
Primary school	1771	43.1	346	46.7	365	50.1	
Middle school	480	11.7	84	11.3	75	10.3	
High school	1065	25.9	125	16.9	124	17.0	
More than high school	442	10.8	41	5.5	50	6.9	
Missing	2		2		0		

Supplementary Figure S1. Flow chart of data for analysis

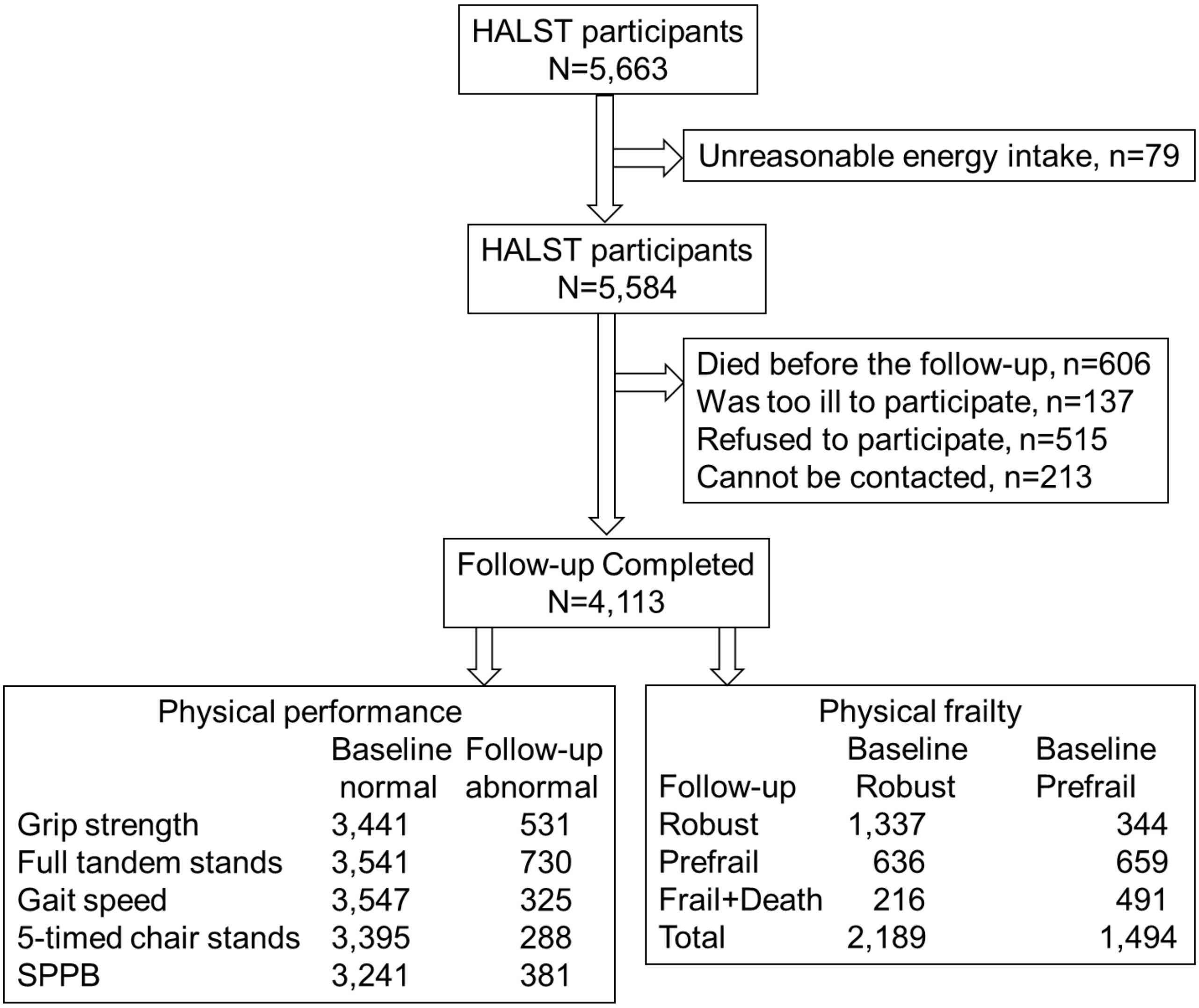

Supplement: Supplementary file 1 [file nutrients-16-02862-s001.zip › nutrients-3151425-supplementary.pdf]
